# Coexisting Optic Pathway and Posterior Fossa Gliomas in an Adult With Previously Undiagnosed Neurofibromatosis Type 1

**DOI:** 10.7759/cureus.104747

**Published:** 2026-03-05

**Authors:** Clara Pinto, Ana P Santos, João Faia, Inês S Pinheiro, Ana S Martins

**Affiliations:** 1 Internal Medicine, Unidade Local da Região de Aveiro, Aveiro, PRT

**Keywords:** café-au-lait macules, central nervous system neoplasm, neurofibromatosis type 1, optic pathway glioma, posterior fossa tumors

## Abstract

Neurofibromatosis type 1 (NF1) is a common autosomal dominant disorder that predisposes to central nervous system (CNS) gliomas. While pediatric optic pathway gliomas (OPGs) are typically benign, adult non-optic pathway gliomas (non-OPGs) represent a distinct entity, often with aggressive behavior and poorer prognosis. We report a previously undiagnosed 49-year-old woman presenting with progressive ataxia and vertical diplopia. Clinical evaluation revealed multiple café-au-lait macules, cutaneous neurofibromas, and bilateral Lisch nodules, confirming the diagnosis of NF1. Neuroimaging demonstrated a coexisting OPG and a symptomatic expansile non-OPG in the posterior fossa and cerebellar peduncle. Symptomatic onset and cerebellar involvement are adverse prognostic factors. Adult non-OPGs are associated with aggressive clinical courses, with reported median overall survival around 24 months, regardless of histological grade. This case emphasizes the importance of recognizing cutaneous and ophthalmological features for timely NF1 diagnosis. Adult non-OPGs warrant extended radiological surveillance, particularly in high-risk CNS locations, due to their potential for aggressive behavior.

## Introduction

Neurofibromatosis type 1 (NF1), also known as von Recklinghausen disease, is a common autosomal dominant disorder and the most frequent neurocutaneous syndrome [1], with an estimated incidence of approximately one in 3,000 individuals [2]. NF1 is a multisystem disease characterized by cutaneous manifestations, including café-au-lait macules, axillary or inguinal freckling, and multiple cutaneous neurofibromas, which represent key components of the National Institutes of Health (NIH) diagnostic criteria [2,3]. Patients with NF1 also have an increased risk of developing central nervous system (CNS) tumors, particularly gliomas [1].

NF1-associated gliomas occur predominantly in childhood, with optic pathway gliomas (OPGs) representing the most common intracranial neoplasm, affecting approximately 15-20% of pediatric patients and typically following a benign clinical course [3,4]. In contrast, non-optic pathway gliomas (non-OPGs) represent a distinct clinical entity, and the synchronous occurrence of optic pathway gliomas and non-OPGs in adulthood has been reported only rarely [1]. These tumors are more frequently located in non-classical CNS regions, including the brainstem, thalamus, basal ganglia, cerebellum and corpus callosum, and tend to present symptomatically [3,4].

Current evidence suggests that adult-onset, non-optic pathway gliomas in NF1 are associated with worse clinical outcomes compared with pediatric OPGs, even when histologically classified as low-grade, according to the WHO Classification of Tumors of the Central Nervous System [4,5]. Tumor location outside the optic pathways, diagnosis in adulthood, and symptomatic presentation have been identified as adverse prognostic factors, increasing the risk of delayed diagnosis and neurological morbidity, particularly in patients without a prior diagnosis of NF1 [1,4].

We report a rare case of an adult patient with previously undiagnosed NF1 whose first clinically significant manifestation was the synchronous coexistence of an optic pathway glioma and a posterior fossa glioma. This case highlights the importance of considering NF1 in adults presenting with atypical CNS tumor patterns and recognizing cutaneous features to avoid missed or delayed diagnosis [2,6].

This case was presented as an oral presentation at the 28th National Congress of Internal Medicine, in Portugal, in October 2022.

## Case presentation

A 49-year-old woman with a history of arterial hypertension presented to the emergency department with progressive nausea and greenish watery vomiting lasting approximately one month, unrelated to food intake and refractory to antiemetic therapy. She also reported a two-month history of vertical binocular diplopia and, more recently, a left frontotemporal headache of fluctuating intensity. There were no focal motor deficits, cognitive impairment, or significant visual loss at presentation. A family history suggestive of a similar but undiagnosed condition was noted in her mother, who was deceased.

**Figure 1:**
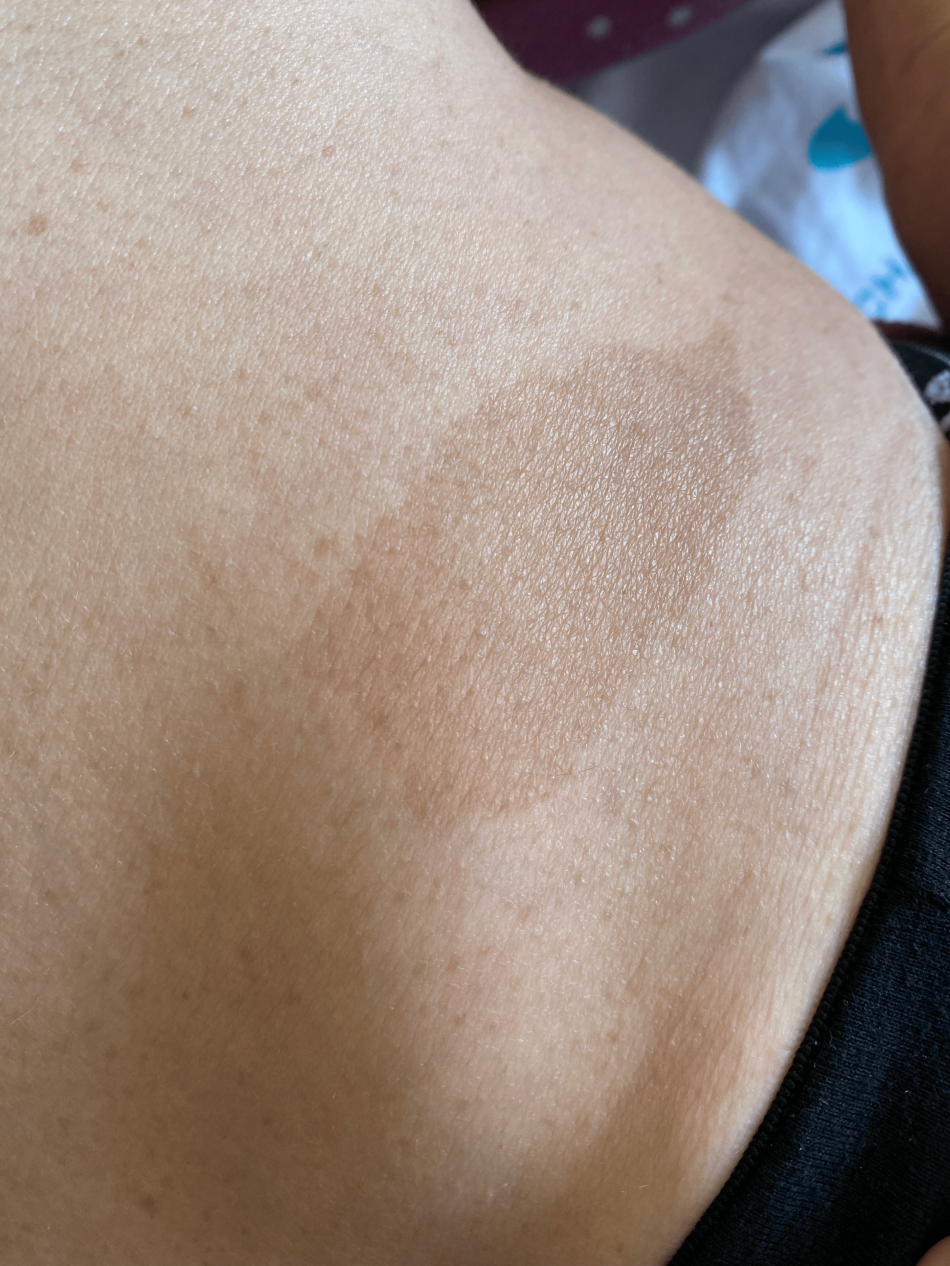
Café-au-lait macules.

**Figure 2:**
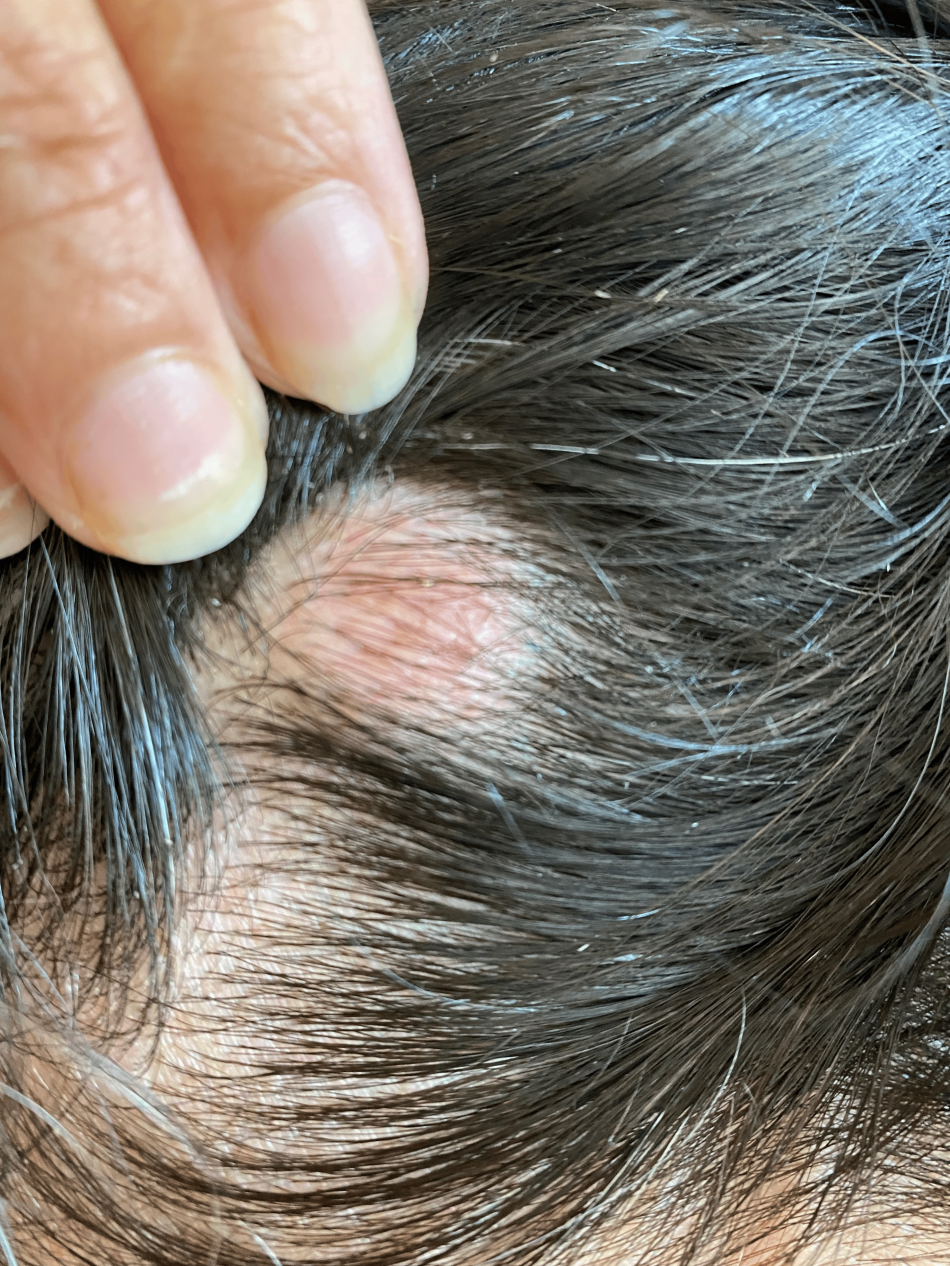
Cutaneous neurofibromas on the scalp.

**Figure 3:**
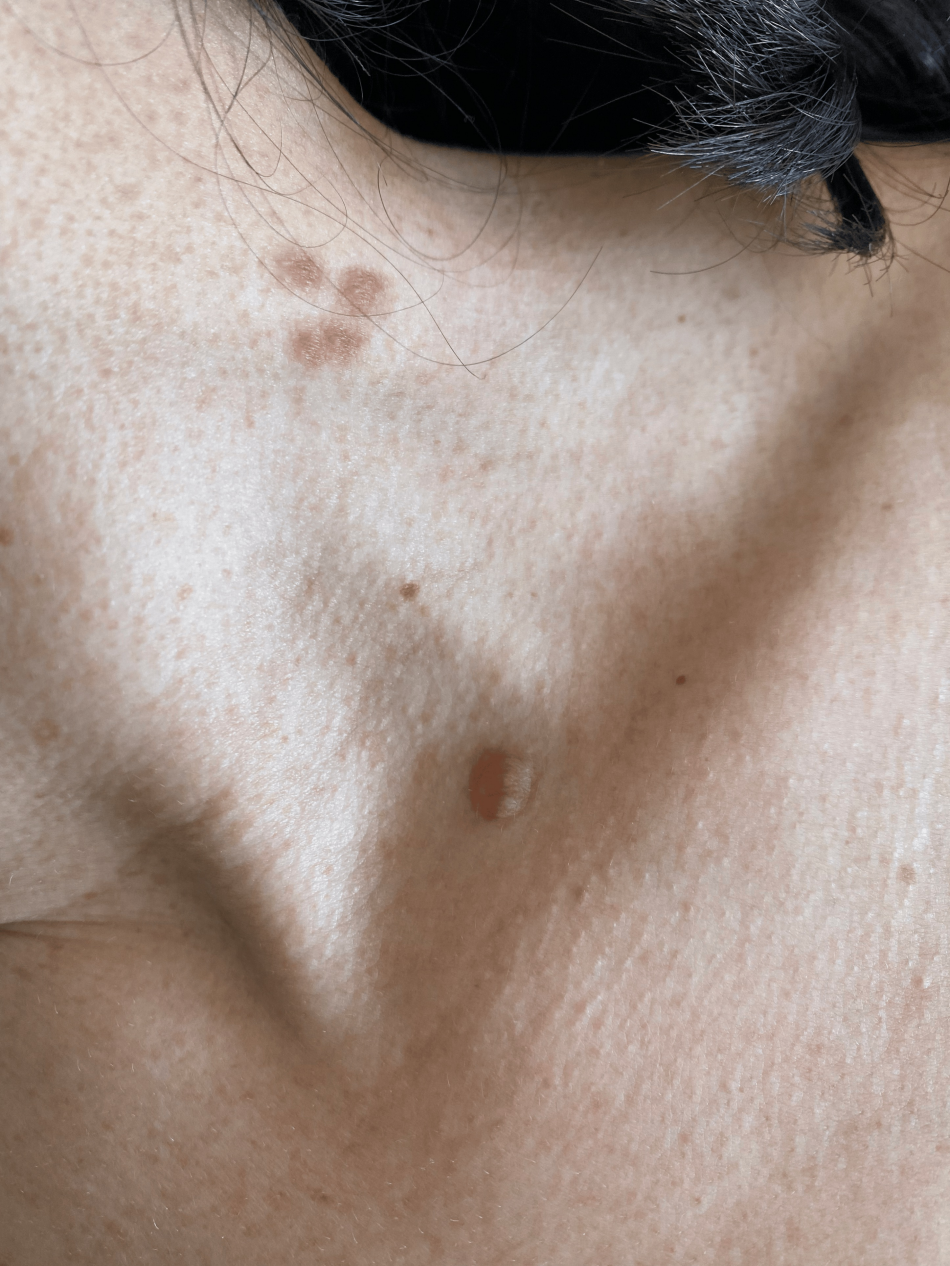
Cutaneous neurofibromas on the cervical region.

Physical examination revealed multiple café-au-lait macules (Figure 1) distributed over the body, as well as cutaneous neurofibromas on the scalp (Figure 2) and cervical region (Figure 3). Neurological examination demonstrated vertical strabismus, spontaneous and gaze-evoked nystagmus, and axial and right-sided limb ataxia.

**Figure 4:**
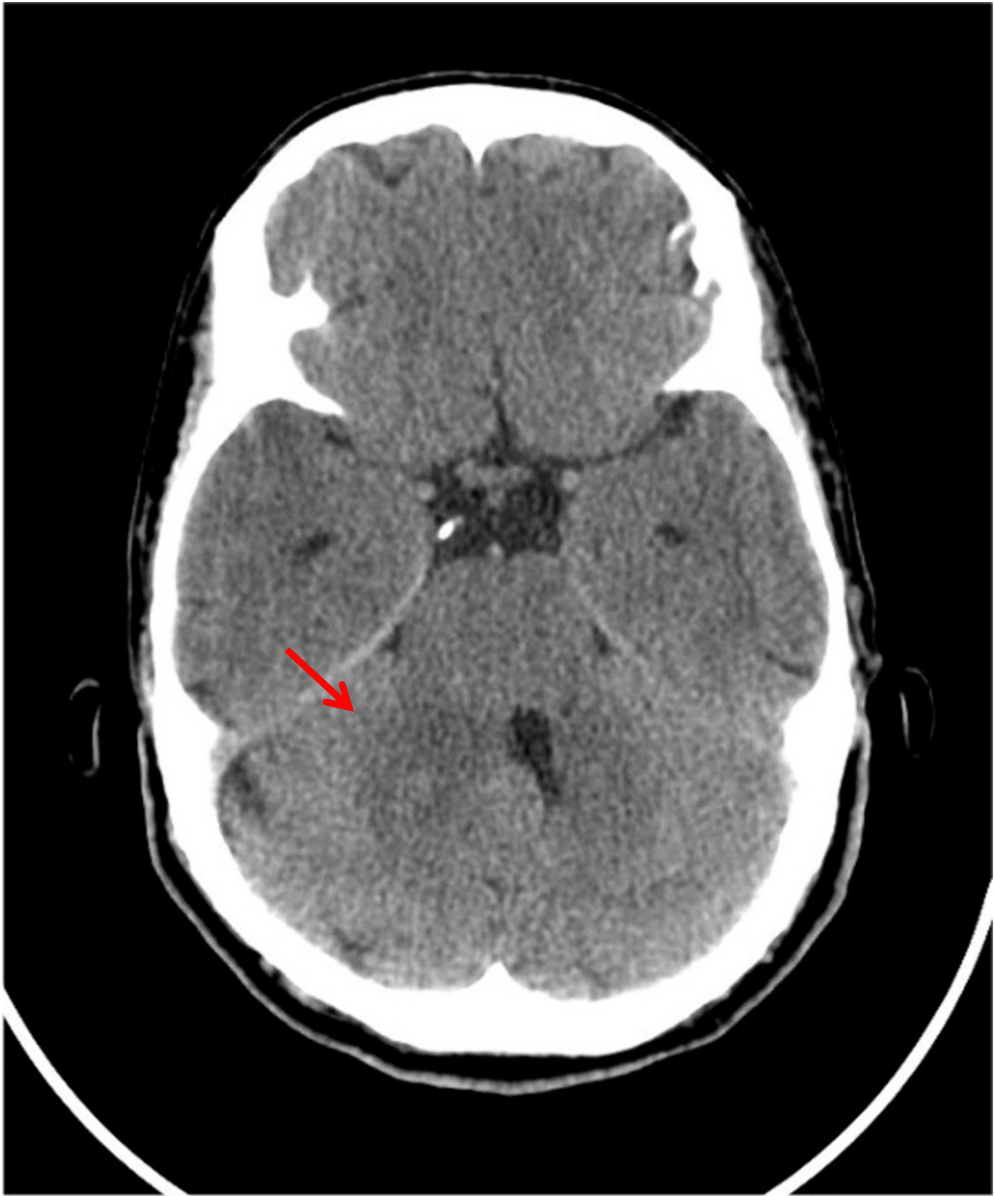
Cranial computed tomography showing a space-occupying lesion involving the right cerebellar hemisphere (red arrow).

**Figure 5:**
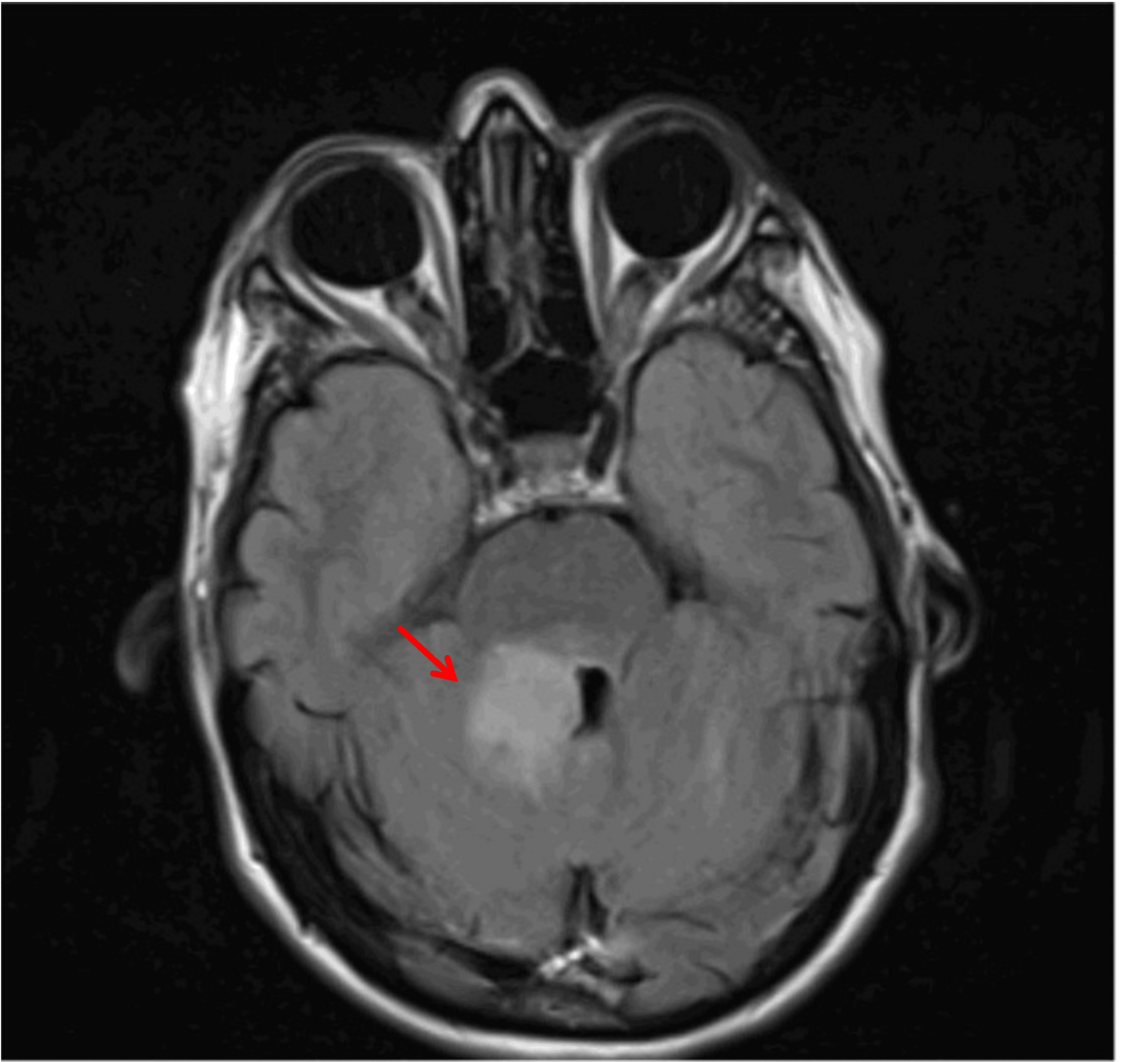
Brain magnetic resonance imaging (T2-FLAIR sequence) showing an expansile lesion (red arrow) of the right cerebellar peduncle with posterior fossa involvement. FLAIR: fluid-attenuated inversion recovery.

Cranial computed tomography revealed a space-occupying lesion involving the middle cerebellar peduncle and the superior aspect of the right cerebellar hemisphere (Figure 4). Brain magnetic resonance imaging confirmed an expansile lesion centered in the right middle and superior cerebellar peduncle (measuring approximately 24.4 mm vertical x 18.6 mm transversal x 13.7 mm anteroposterior), extending to the right posterolateral pontomesencephalic junction, associated with mild surrounding edema, fourth ventricle molding, and posterior fossa involvement (Figure 5). Enlargement of the optic nerves along their intraorbital and prechiasmatic intracranial course was also observed. The patient was evaluated by Neurosurgery and Ophthalmology. A compressive left optic neuropathy was confirmed, along with the bilateral presence of Lisch nodules on iris examination. The constellation of clinical, cutaneous, ophthalmological, and radiological findings raised a strong suspicion of neurofibromatosis type 1 with CNS involvement [2,3]. Histopathological examination of the posterior fossa lesion biopsy confirmed the diagnosis of glioma.

Due to clinical deterioration associated with worsening hydrocephalus and reduced patency of the prepontine cisterns, an external ventricular drain was placed in July 2022. Subsequently, in July 2022, the patient underwent endoscopic ventriculostomy. Following a multidisciplinary discussion, a palliative therapeutic approach was adopted due to progressive tumor growth. The patient was started on dexamethasone at a dose of 8 mg and was referred for whole-brain radiotherapy.

Radiotherapy was initiated in April 2023 but was prematurely discontinued within nine days due to a significant worsening of her general condition, resulting in a total dose of 21 Gy instead of the initially planned 30 Gy. Following discontinuation of radiotherapy, the patient remained under supportive care and follow-up by her neurologist, as she was no longer considered fit for further oncological treatment. She died in April 2023.

## Discussion

NF1 is associated with a highly increased risk of developing CNS neoplasms, with gliomas being the most frequent [6]. Classically, these tumors occur predominantly in childhood, with OPGs representing the most common intracranial neoplasm in pediatric patients, typically following a benign clinical course [4]. However, in adulthood, NF1-associated gliomas that develop outside the optic pathways (non-OPGs) constitute a distinct clinical entity [4]. These tumors tend to arise in less typical or non-classical CNS locations, frequently involving midline structures such as the brainstem, cerebellum (posterior fossa), thalamus, basal ganglia, and corpus callosum [4].

This case is notable for the coexistence of an OPG (evidenced by enlarged optic nerves and confirmed optic neuropathy) and a non-OPG (posterior fossa glioma) in a 49-year-old patient without a prior diagnosis of NF1 [2,4]. While multiple CNS gliomas are relatively common in patients with known NF1 [6], the synchronous presentation of optic pathway and posterior fossa tumors in adulthood is exceedingly rare. Pediatric OPGs often remain stable or regress; however, OPGs diagnosed in adulthood may be active and demonstrate clinical progression and visual deterioration in a significant proportion of cases, a pattern mirrored by this patient’s rapid visual decline [7]. To our knowledge, such a presentation as the primary clinical manifestation of previously undiagnosed NF1 has only been reported exceptionally [4,8].

In contrast to many reported cases in which NF1 is diagnosed in childhood based on dermatological manifestations, this patient remained asymptomatic from a syndromic standpoint until the fifth decade of life. This late-stage diagnosis aligns with reports suggesting that adult-diagnosed NF1 patients may harbor a distinct molecular profile or a lower mutational burden that delays tumor onset; however, once tumors develop, they may bypass the indolent phase typically observed in pediatric cases [9].

The absence of a prior NF1 diagnosis and the predominance of symptoms attributable to the tumor's anatomical location (e.g., nausea, vomiting, ataxia, diplopia) may contribute to delayed recognition [1,4]. Importantly, the presence of glioma-related symptoms at diagnosis is recognized as an adverse prognostic factor associated with increased mortality [4]. Tumor location is also highly relevant, as involvement of the cerebellum and brainstem is associated with a higher likelihood of requiring surgical intervention [1]. Unlike the cases reported by Byrne et al., in which many non-OPGs were incidental findings detected through surveillance imaging, our patient’s tumor was symptomatic at presentation, a factor that correlates with higher histological grade and poorer survival outcomes [1,10].

This clinical presentation underscores the prognostic challenges associated with non-OPGs in adults with NF1. Although low-grade gliomas are generally indolent, non-OPGs diagnosed in adulthood-even those histologically classified as WHO grade 1 or 2 [5]-may follow an aggressive clinical course [4]. This discrepancy may be explained by the accumulation of secondary genetic alterations, such as CDKN2A deletions or TP53 mutations, which are more prevalent in adult NF1-associated gliomas than in pediatric cases [10]. In the present case, the lesion demonstrated aggressive behavior, leading to palliative radiotherapy; nevertheless, the disease progressed rapidly, and the patient died shortly thereafter, highlighting the poor prognosis associated with symptomatic non-OPGs in the adult population. This aggressive course reinforces the importance of early syndromic recognition.

This case further emphasizes the importance of meticulous clinical examination, particularly the identification of cutaneous and ophthalmological features-multiple café-au-lait macules, cutaneous neurofibromas, and Lisch nodules-which fulfilled the NIH diagnostic criteria for NF1 [2]. Recognition of these findings was essential for establishing a unifying syndromic diagnosis and guiding management [2]. Given the aggressive potential and unpredictable clinical behavior of non-OPGs in adulthood, particularly in critical locations such as the thalamus, intensified surveillance with more frequent imaging should be considered [1]. In general, serial imaging for at least five years after tumor detection is recommended, as 91% of patients requiring surgical intervention do so within this period [1].

## Conclusions

In adults, NF1 may present with CNS tumors in atypical locations (non-OPGs) beyond the optic pathways. As demonstrated in this case, the coexistence of an OPG and a posterior fossa glioma in a previously undiagnosed adult can be the sentinel manifestation of the disease. Therefore, a high index of suspicion for NF1 should be maintained in adults presenting with multiple or atypically located CNS gliomas, as early syndromic recognition has critical prognostic and surveillance implications.

Establishing a confirmed syndromic diagnosis is essential, as the symptomatic presentation and tumor location (e.g., cerebellum, midline structures) are often associated with a potentially worse clinical outcome and require prolonged radiological surveillance for at least five years.

## References

[1] Byrne S, Connor S, Lascelles K, Siddiqui A, Hargrave D, Ferner RE (2017). Clinical presentation and prognostic indicators in 100 adults and children with neurofibromatosis 1 associated non-optic pathway brain gliomas. J Neuro-oncol.

[2] Le C, Thomas A, Lui F (2025). Neurofibromatosis. StatPearls (Internet).

[3] Friedman JM (1998). Neurofibromatosis 1. GeneReviews (Internet).

[4] Romo CG, Piotrowski AF, Campian JL (2023). Clinical, histological, and molecular features of gliomas in adults with neurofibromatosis type 1. Neuro Oncol.

[5] Louis DN, Perry A, Wesseling P (2021). The 2021 WHO classification of tumors of the central nervous system: a summary. Neuro Oncol.

[6] Barbosa M, Manso M, Carvalho L (2011). Neurofibromatosis type 1-unpredictable disease?. Rev Port Med Interna.

[7] Shofty B, Constantini S, Bokstein F (2014). Optic pathway gliomas in adults. Neurosurgery.

[8] Sellmer L, Farschtschi S, Marangoni M (2017). Non-optic glioma in adults and children with neurofibromatosis 1. Orphanet J Rare Dis.

[9] Pemov A, Hansen NF, Sindiri S (2019). Low mutation burden and frequent loss of CDKN2A/B and SMARCA2, but not PRC2, define premalignant neurofibromatosis type 1-associated atypical neurofibromas. Neuro Oncol.

[10] Lucas CG, Sloan EA, Gupta R (2022). Multiplatform molecular analyses refine classification of gliomas arising in patients with neurofibromatosis type 1. Acta Neuropathol.

